# Effect of Trehalose and Trehalose Transport on the Tolerance of* Clostridium perfringens* to Environmental Stress in a Wild Type Strain and Its Fluoroquinolone-Resistant Mutant

**DOI:** 10.1155/2016/4829716

**Published:** 2016-12-12

**Authors:** Miseon Park, Wilfrid J. Mitchell, Fatemeh Rafii

**Affiliations:** ^1^Division of Microbiology, National Center for Toxicological Research, FDA, Jefferson, AR 72079, USA; ^2^School of Life Sciences, Heriot-Watt University, Riccarton, Edinburgh EH14 4AS, UK

## Abstract

Trehalose has been shown to protect bacterial cells from environmental stress. Its uptake and osmoprotective effect in* Clostridium perfringens *were investigated by comparing wild type* C. perfringens* ATCC 13124 with a fluoroquinolone- (gatifloxacin-) resistant mutant. In a chemically defined medium, trehalose and sucrose supported the growth of the wild type but not that of the mutant. Microarray data and qRT-PCR showed that putative genes for the phosphorylation and transport of sucrose and trehalose (via phosphoenolpyruvate-dependent phosphotransferase systems, PTS) and some regulatory genes were downregulated in the mutant. The wild type had greater tolerance than the mutant to salts and low pH; trehalose and sucrose further enhanced the osmotolerance of the wild type to NaCl. Expression of the trehalose-specific PTS was lower in the fluoroquinolone-resistant mutant. Protection of* C. perfringens* from environmental stress could therefore be correlated with the ability to take up trehalose.

## 1. Introduction

*Clostridium perfringens,* a Gram-positive, spore-forming anaerobic bacterium, produces several different toxins. Although a pathogen, it is a member of the normal gastrointestinal tract microbiota of humans and animals and is found in soil, sewage, and foods [1]. Its ubiquitous distribution indicates that* C. perfringens* can overcome environmental stresses to survive. Forming spores allows* C. perfringens* to cope with harsh environmental conditions [2]. A two-component signal transduction system, VirR and VirS, enables* C. perfringens* to sense environmental changes and regulate the transcription of genes for the expression of the appropriate response [3].

In addition, bacteria may accumulate protective compounds, such as osmolytes, which prevent bacterial damage under stressful conditions by sheltering macromolecules and membranes from an otherwise harmful environment [4–9]. The disaccharides trehalose and sucrose are among these osmolytes that protect cells from environmental stress [5, 6, 10–15]. Increased intracellular trehalose has been reported in bacteria after treatment with NaCl [12, 16]. 1 mM trehalose has been shown to protect bacteria against 0.5 M NaCl [14]. Although the protective role of sucrose has been shown during L-form formation in* C. perfringens* [17], the role of trehalose in the protection of* C. perfringens* from environmental stress is not known.* Escherichia coli* and some other bacteria have enzymes for trehalose biosynthesis [4, 12, 13, 18–20] but no putative gene for trehalose synthesis has been detected in the genomes of strains of* C. perfringens* that have been sequenced.

In clostridia, like other bacteria, sugars may be transported into the cell via the phosphoenolpyruvate-dependent sugar phosphotransferase system (PTS), which both transports and phosphorylates the substrate [21–23]. Based on the structural similarities to enzymes of the PTS IIBC subunit sugar transport genes, a putative gene for transport and metabolism of trehalose via the PTS has been identified in* C. perfringens* [2, 24]. Three genes (*treB*,* treC,* and* treR*) have been annotated as involved in trehalose transport, metabolism, and regulation [2, 24]. As in other bacteria, the* treB* gene product is proposed to transport trehalose into the cell as trehalose 6-phosphate, which is then hydrolyzed via the* treC* gene product *α*,*α*-phosphotrehalase (trehalose-6-phosphate hydrolase), to produce glucose 6-phosphate and glucose [18, 25]. In* Bacillus subtilis*, both* treR* and carbon catabolite repression (CCR) regulate the transport and metabolism of trehalose [26]. No trehalose-specific EIIA gene is listed among the genes found in the sequence of* C. perfringens *13124 in GenBank. However, the missing EIIA function may be provided by the EIIA domain of another PTS, as has been demonstrated in the case of the trehalose PTS in* B. subtilis* [27].

In food and in the gastrointestinal tract,* C. perfringens* may come in contact with trehalose synthesized by other organisms [9] and also may be exposed to antimicrobial agents used for the treatment of infections [28, 29]. Exposure to antimicrobial agents may result in the development of resistant bacterial strains with changes that affect their metabolic activities [30–32]. We have shown strain-specific changes in fluoroquinolone-resistant strains of* C. perfringens* generated in the laboratory [28, 32–34]. In gatifloxacin-resistant laboratory mutants of* C. perfringens* ATCC 13124 [35], the expression of various genes and production of toxins has been altered [28]. In this study, we have investigated other alterations in the mutant by comparing the growth of mutant and wild type* C. perfringens* under various conditions and the role of trehalose in protecting them from environmental stress. We found that the genes designated as* treB *and* treC* are functionally involved in trehalose utilization and that their expression and their role in protection of cells from environmental stress have been altered in one fluoroquinolone-resistant mutant.

## 2. Materials and Methods

### 2.1. Growth of Strains

*Clostridium perfringens* ATCC 13124 (wild type) and its fluoroquinolone-resistant mutant 13124^GR^, which has a stable double mutation in g*yrA *[35], were used for the experiment. The mutant was developed with exposure to 10 *µ*g/ml of gatifloxacin [35]. The susceptibility of the mutant to different fluoroquinolones, including gatifloxacin, varied 128-fold [35]. The resistance development affected the expression of various genes [28]. The bacteria were grown in an anaerobic glove box (Coy Laboratory Products, Inc.) under 85% N_2_, 10% CO_2_, and 5% H_2_, in brain heart infusion (BHI) medium for all experiments unless otherwise stated.

### 2.2. Assay for Growth on Different Sugars in Minimal Medium and BHI

The minimal medium, in phosphate buffer, included 19 amino acids, uracil, adenine, vitamins (biotin, calcium D-pantothenate, riboflavin, and pyridoxamine), and salts [36]. The chemically defined media, with and without sugars, were filter-sterilized. To assay strains for the ability to metabolize glucose, fructose, galactose, sucrose, and trehalose, a final concentration of 1% of one of the sugars was added to the minimal medium. The minimal medium without sugar was used for controls. The media were dispensed into triplicate wells in 96-well microtiter plates, inoculated with washed cells of overnight cultures of the wild type and the mutant, and incubated at 37°C under anaerobic conditions. Growth was measured spectrophotometrically (Biotek Instruments) at 600 nm after 24 h.

The effect of trehalose on growth was also examined in brain-heart infusion (BHI) medium. Heat sterilized BHI with and without trehalose (at concentrations up to 5%) was placed in triplicate wells of microtiter plates and inoculated with the mutant and wild type. Growth was examined after the plates were incubated at 37°C for 24 h under anaerobic conditions.

### 2.3. PCR Analysis

The sequence of* C. perfringens* ATCC 13124 from GenBank (accession number CP000246) was used to design primers for the amplification of genes involved in the metabolism of carbohydrates. The primers were designed to amplify the genes for trehalose utilization,* treB, treC*, and* treR*, the flanking sequences upstream and downstream of these genes and some regulatory genes (Table 1); DNA Star software was used to design primers. DNA was extracted according to a method described previously [35]. The 100 *μ*l PCR mixture contained template DNA, 2 mM of dNTP mix, 0.5 *μ*M of each forward and reverse primer, and 2.5 units of Taq polymerase from Roche or Applied Biosystems. The MyCycler thermocycler from Bio-Rad was used to amplify the genes. After initial denaturation at 95°C for 2 min, the genes were amplified during 30 cycles with the following parameters: 95°C for 15 sec, 52°C (or another appropriate temperature for the annealing of primers) for 30 sec, and 72°C for 90 sec, and final extension for 5 min at 72°C. The PCR products were purified from agarose gels and sequenced using an ABI Biosystems sequencer and dideoxy terminator.

### 2.4. Preparation of RNA

Total RNA was isolated from cultures of the wild type and mutant [28]. Briefly, cells were harvested by centrifugation (15,000 ×g, 10 min, 4°C), washed with 10 mM Tris and 1 mM EDTA (pH 8.0), and suspended in buffer containing 1 mg/ml of lysozyme (Sigma) and 2% SDS. RNA Bee (Gentaur, Brussels, Belgium) was added to the mixture and then the RNA was extracted with chloroform. The RNA in the top layer was precipitated with isopropanol by placing the mixture at −70°C for 30 min. After centrifugation, the pellet was drained and washed twice with 75% ethanol and dissolved in diethylpyrocarbonate- (DEPC-) treated water before treatment with DNase I (Ambion, Santa Clara, CA). Qiagen RNeasy columns were used to further purify the RNA according to the manufacturer's instructions.

### 2.5. qRT-PCR for Comparison of Relative Gene Expression in the Wild Type and Mutant Strain

qRT-PCR was performed using the SYBR®GreenER™ qPCR SuperMix Universal Two-Step qRT-PCR kit (Invitrogen) according to the manufacturer's instructions, using the primers listed in Table 2. Primers for 16S rRNA were used as controls. In the first step of qRT-PCR, cDNA was synthesized from RNA using the SuperScript™ III First-Strand Synthesis SuperMix (Invitrogen) component of the kit and 25 ng/*μ*l of RNA, in reaction mixtures with and without the RT enzyme. The cDNA was cooled and mixed with* E. coli* RNase H, provided in the kit, and incubated for 20 min at 37°C. The cDNA was diluted and amplified in the second step of qRT-PCR, using the SYBR®GreenER™ qPCR SuperMix component of the kit. The C1000 thermal cycler CFX96 real-time system from Bio-Rad was used for the synthesis with the following parameters: 50°C for 2 min, 95°C for 8.5 min, and 40 cycles of 95°C for 15 sec and 60°C for 30 sec. The instrument was programmed to measure the melting curve within 65–95°C (1°/5 sec). The Ct (cycle threshold) for each of the genes was determined. The fold change in gene expression was calculated for each of the genes in the mutant and wild type [28]. The 2^−ΔΔC^T method was used to calculate the relative level of mRNA expression, according to the thermocycler application guide [28].

### 2.6. Effect of Sodium Chloride on the Bacterial Growth in the Presence and Absence of Trehalose

NaCl concentrations of 0–2% were added to triplicate wells of 96-well microtiter plates containing BHI, with or without trehalose (0.5%). The wells were inoculated with the wild type and mutant. To find out the effect of trehalose in protection of cells from NaCl, the kinetics of the growth of wild type* C. perfringens* ATCC 13124 in the presence of 1% glucose, sucrose, or trehalose and also combinations of 1% glucose + 1% trehalose and 1% glucose + 1% sucrose were measured. 0.5 ml aliquots of minimal media supplemented with sugars were inoculated with overnight cultures of* C. perfringens* ATCC 13124, in the presence of 0%, 0.5%, 1%, and 2% NaCl in 48-well microtiter plates. The wells were filled with 0.9 ml of sterile mineral oil to keep the cultures anaerobic. The plates were placed in the Synergy Max Spectrophotometer (Biotek Instruments) and the kinetics module of Gene 5 software (Biotek Instruments) was programmed to measure the A_600_ every 30 min at 37°C. The kinetics analysis was performed using Microsoft Excel.

### 2.7. Effect of Sodium Nitrite on Growth in the Presence and Absence of Trehalose

NaNO_2_ concentrations of 0–0.8% were added to triplicate wells of 96-well microtiter plates containing BHI with and without 0.5% trehalose. The wells were inoculated with the wild type or mutant and incubated anaerobically at 37°C. Growth was monitored at 600 nm with a spectrophotometer.

### 2.8. Effect of pH on the Growth of Wild Type and Mutant in the Presence and Absence of Trehalose

BHI medium, with pH values ranging within 4.8–10, was prepared and dispensed in triplicate wells of two 96-well microtiter plates. In one of the plates, trehalose (0.5%) was added to the wells. The plates were inoculated with overnight cultures of wild type and mutant and incubated anaerobically at 37°C. Growth was measured spectrophotometrically at 600 nm after 24 h.

## 3. Results

### 3.1. Growth of* C. perfringens* on Different Sugars in Minimal Medium and BHI

**Figure 1:**
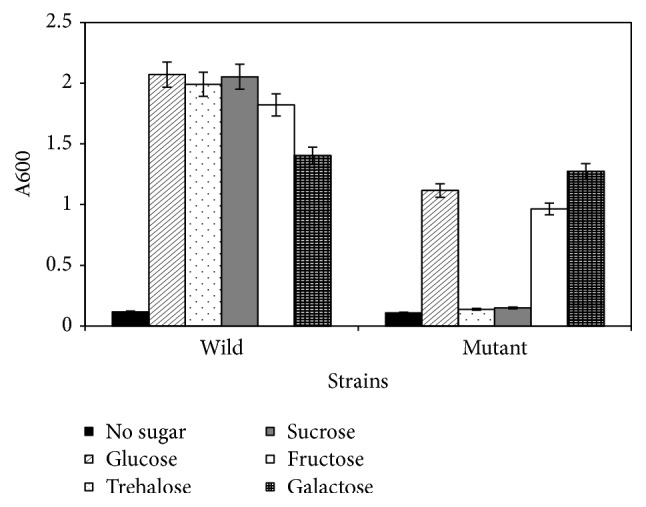
Growth of wild type* C. perfringens* ATCC 13124 and the mutant 13124^GR^ in minimal medium with different sugars. Error bars = standard deviation.

To find out if there were differences between the mutant and wild type in metabolizing different sugars, bacteria were grown in both minimal and rich media containing various sugars. The wild type ATCC 13124 grew in defined minimal medium containing glucose, sucrose, trehalose, fructose, or galactose (Figure 1). The mutant 13124^GR^ had less growth than the wild type on glucose, fructose, and galactose and did not grow on trehalose or sucrose (Figure 1).

**Figure 2:**
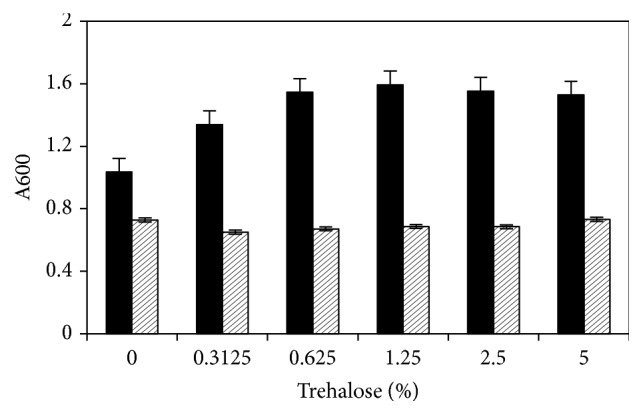
Effect of different concentrations of trehalose on the growth of wild type* C. perfringens *ATCC 13124 (black bars) and mutant 13124^GR^ (cross-hatched bars) in brain heart infusion (BHI).

In BHI medium supplemented with sugars, both strains grew (Figure 2). Addition of different concentrations of trehalose to BHI resulted in increased growth of the wild type, but only up to 1.25% trehalose. Addition of trehalose to BHI cultures of the mutant 13124^GR^ had no effect on growth (Figure 2). Gatifloxacin resistance affected the expression of various genes, decreasing the fitness of the 13124^GR^ resistant mutant in comparison to the wild type [28, 32]. Therefore, the resistant mutant had less growth than the wild type, both in BHI medium and in the minimal medium with sugars that supported its growth.

### 3.2. Sequence Analysis and Expression of Genes Involved in Trehalose Transport and Metabolism and Regulatory Genes

To find out if the lack of ability of the mutant to grow on trehalose was because of mutation in the annotated trehalose transport gene or related PTS regulatory genes, these genes were amplified by PCR and sequenced. The primers used for the amplification of genes of the trehalose transport operon amplified the same size fragments from* treB*,* treC*, and* treR* in DNA from both mutant and wild type. The sequences of the amplified genes for* treB*, a trehalose-specific IIBC component of the PTS;* treC*, trehalose-6-phosphate hydrolase;* treR*, a transcriptional regulator for these genes; and the flanking regions were identical for the mutant and wild type with one exception: a mutation of G^368^T was observed in* treB* that resulted in the substitution of Leu 123 with Trp.

Other known PTS and global regulatory genes from* C. perfringens* were also sequenced. The sequences of the HPr kinase/phosphorylase (HPrK, CPF_1261), the catabolite control protein A (CcpA, CPF_2863), and the sigma factor 70 (*σ* ^70^, CPF_0539) that are involved in PTS-related regulatory functions were identical for the wild type and the mutant. The sequence of the global regulatory gene* vrr*, which regulates a variety of genes in* C. perfringens*, was also identical in the wild type and mutant. However, a PTS regulatory gene (CPF_0069), which codes for a transcription antiterminator, had a single mutation resulting in the conversion of Leu 90 to Ser.

### 3.3. Comparison of the Transcription of Genes Involved in Trehalose Transport and Regulatory Genes

To find out if there were differences in expression of genes involved in trehalose transport in wild type and the mutant, the results of microarray data and gene transcription, as measured by qRT-PCR, of these genes in the two strains were compared. Microarray analysis data (http://www.ebi.ac.uk/arrayexpress/arrays/A-MEXP-2008/) showed alteration of the transcription of a range of genes in the mutant 13124^GR^ compared to the wild type [28]. Among the downregulated genes were* treB* and* treC*, which are involved in trehalose transport and metabolism. The qRT-PCR experiment, performed to compare the expression of several genes in the wild type and mutant, confirmed the microarray data on the downregulation of expression of* treB *and* treC* in the mutant 13124^GR^. The transcription of the gene encoding the IIBC component of the putative sucrose PTS was also downregulated (Table 2). In addition, expression of the global regulatory gene* vrr* (CPF_1204) and a PTS-associated antiterminator gene (CPF_0069) were also decreased substantially in the mutant 13124^GR^. CPF_0069 is located upstream of the putative PTS* N*-acetylglucosamine-specific EIIA and EIIBC operon; the expression of those genes was also downregulated.

### 3.4. Growth of the Mutant and Wild Type with Sodium Chloride

**Figure 3:**
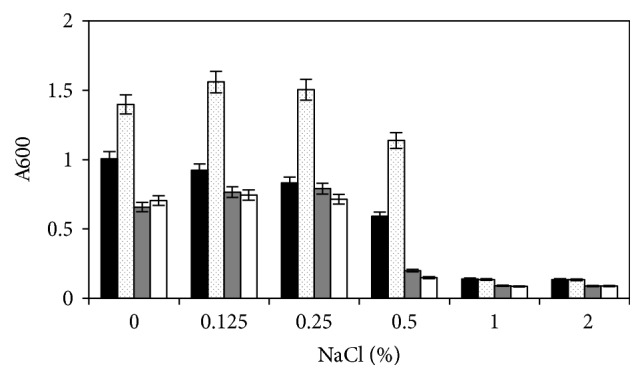
Growth of wild type* C. perfringens *ATCC 13124 and mutant 13124^GR^ with different concentrations of NaCl in the absence and presence of trehalose. Black bars (wild type without trehalose), gray bars (mutant without trehalose), dotted bars (wild type with trehalose), and white bars (mutant with trehalose). OD was measured after 24 hours' anaerobic incubation at 37°C.

To find out if there were differences between the NaCl tolerance of mutant and wild type and whether trehalose protects* C. perfringens* from the effect of high salt concentration, the strains were grown with different concentrations of NaCl, with or without trehalose. In BHI medium, the growth of the mutant 13124^GR^ was less than that of the wild type (Figure 3). Addition of up to 0.25% NaCl did not affect the growth of either strain substantially, but unlike the wild type ATCC 13124 the mutant 13124^GR^ could not grow with added 0.5% NaCl. BHI medium, in addition to proteinaceous ingredients, contains NaCl and other sodium salts. It is more viscous than the minimal medium and the actual concentration of salt in this medium is higher than 0.5%. Addition of trehalose increased the tolerance of the wild type to NaCl but did not affect the tolerance of the mutant (Figure 3).

**Figure 4:**
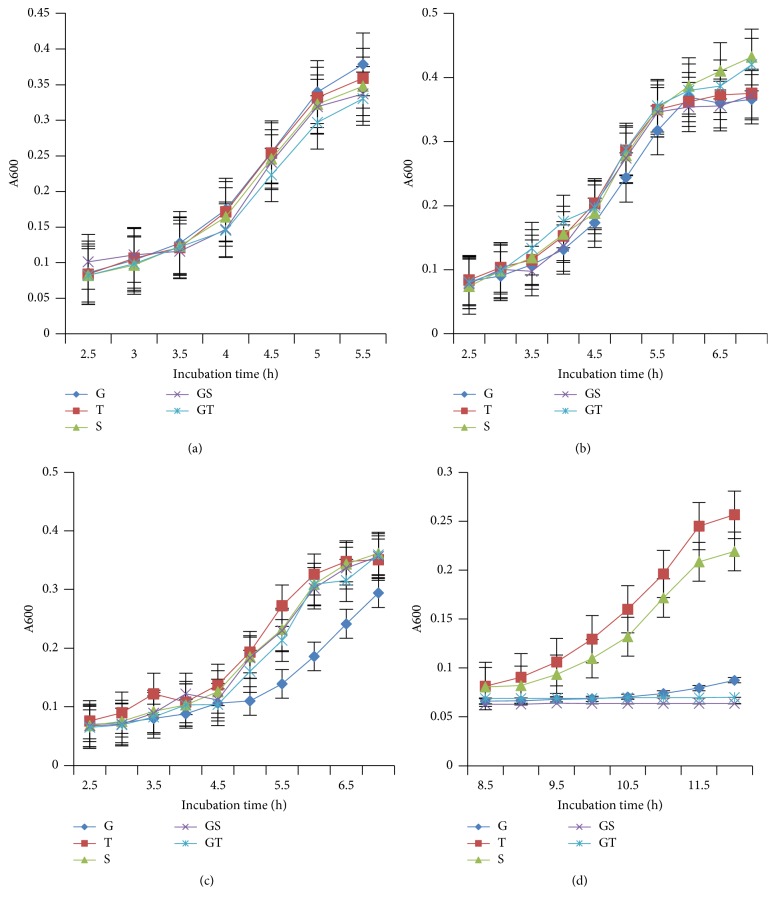
Growth rate of wild type* C. perfringens *ATCC 13124 with different concentrations of NaCl in the presence and absence of trehalose and sucrose in the minimal medium. Without NaCl (a), 0.5% NaCl (b), 1% NaCl (c), and 2% NaCl (d). G = glucose, T = trehalose, S = sucrose, GS = glucose + sucrose, and GT = glucose + trehalose.

To confirm that trehalose protected* C. perfringens* against NaCl, the kinetics of growth of ATCC 13124 in the minimal medium with glucose, sucrose, and trehalose were measured, separately and in combination, in the presence of 0, 0.5, 1, and 2% NaCl (Figure 4). The growth rates of the wild type were the same for all sugars in the absence and the presence of 0.5% NaCl (Figure 4). In the presence of 1% NaCl, the rate of growth in the culture containing glucose had decreased in comparison with the growth in cultures with trehalose or sucrose by 42% and 38%, respectively. Cultures containing trehalose had the highest rate of growth, indicating a trehalose osmoprotective effect; sucrose was also osmoprotective but to a lesser extent. The osmoprotective effect of trehalose was also greater than sucrose in cultures containing 2% NaCl, in which, after an extended lag phase of 8 h, bacteria grew in cultures containing trehalose or sucrose. The combination of trehalose with glucose or sucrose did not support growth of the wild type in the presence of 2% NaCl. Glucose may have had a repressive effect on the uptake of protective trehalose and sucrose.

### 3.5. Effect of Sodium Nitrite on the Wild Type and Mutant, with and without Trehalose

**Figure 5:**
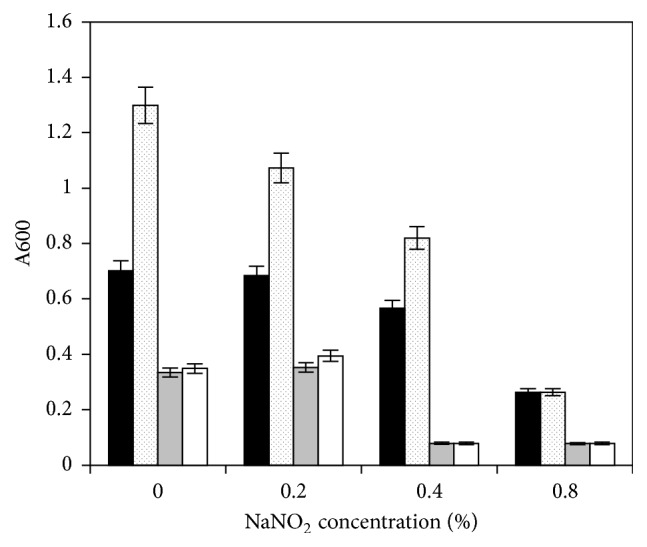
Effect of trehalose on the growth of wild type* C. perfringens *ATCC 13124 and mutant 13124^GR^ in BHI medium in the presence of different concentrations of sodium nitrite. Black bars, wild type without trehalose; dotted bars, wild type with trehalose; gray bars, mutant without trehalose; white bars, mutant with trehalose.

Since NaNO_2_ is used as a preservative in food, its effects on the growth of the wild type and the mutant were also examined in the presence and absence of trehalose. The concentration of NaNO_2_ that inhibited the growth of the wild type ATCC 13124 was twofold higher than that for the mutant 13124^GR^ (Figure 5). Trehalose did not appear to protect either strain against NaNO_2_, but it enhanced the growth of the wild type in both the presence and absence of NaNO_2_ (Figure 5).

### 3.6. Effect of pH on the Growth of Wild Type ATCC 13124 and Mutant 13124^GR^ with and without Trehalose

**Figure 6:**
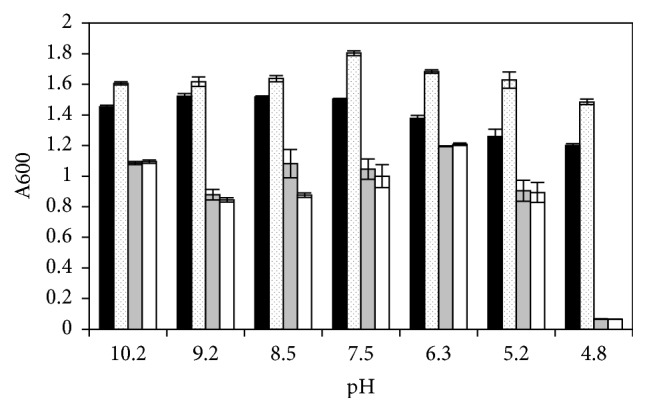
Effect of trehalose on the growth of wild type* C. perfringens *ATCC 13124 and mutant 13124^GR^ at different pH values. Black bars, wild type without trehalose; dotted bars, wild type with trehalose; gray bars, mutant without trehalose; white bars, mutant with trehalose.

Since* C. perfringens* has potential to survive in different environmental conditions, the survival of the strains in the media with different pH was also compared in the presence and absence of trehalose. The wild type ATCC 13124 grew better than the mutant 13124^GR^ in BHI medium at all pH values tested (4.8–10.2) (Figure 6). The lowest pH supporting the growth of the mutant was 5.2, but the wild type could grow in cultures with pH as low as 4.8, both in the presence and in absence of trehalose. Trehalose enhanced the growth of the wild type at acidic to neutral pH, but it had no effect on the growth of the mutant (Figure 6).

## 4. Discussion

We previously generated fluoroquinolone-resistant mutants of* C. perfringens* and showed that resistance development affected the metabolic activities, production of toxins, expression of various genes, and bacterial fitness differently in different strains [28, 32–34]. In this study, we have shown that trehalose was metabolized and had an osmoprotective effect in the wild type ATCC 13124 but not in its fluoroquinolone-resistant mutant 13124^GR^.

The mutant could not grow with sucrose or trehalose in minimal medium and the genes for the transport of these carbohydrates and two regulatory genes, the transcription antiterminator CPF_0069 and* vrr*, were substantially downregulated in the mutant. The mutant was also less tolerant to acidic pH and NaCl. In the BHI medium, trehalose stimulated the growth of the wild type and increased its tolerance to low pH and high salt concentration. In the minimal medium, trehalose had an osmoprotective effect and enabled the bacteria to grow at concentrations up to 2% NaCl. Trehalose is therefore implicated in the protection of the bacteria from environmental stress; and the lack of growth of the mutant 13124^GR^ on trehalose and sucrose in the minimal medium was consistent with inability to transport the sugars into the cell. Although the sequences of* treB*,* treC,* and* treR,* and the region upstream from these genes, which includes the sigma factor *σ* ^70^, were identical except for the mutation in* treB* that resulted in conversion of T^368^G, the transcription of* treB *and* treC *was substantially downregulated, as indicated by both the microarray data and qRT-PCR. Surprisingly, the trehalose transport regulatory gene* treR *(CPF_0543) was not markedly downregulated in the mutant (Table 2).* treR* belongs to the large GntR family of transcriptional regulators; the members of this family regulate varied biological processes.

Trehalose is implicated in the protection of several bacteria from environmental stress [4, 5, 8, 9, 14] and we have shown its osmoprotective effect in* C. perfringens* by demonstrating that addition of trehalose to cultures of ATCC 13124 improved the tolerance of the cultures to increased salt concentration. By comparing the expression of genes proposed to be involved in the transport and metabolism of sucrose and trehalose [2, 24], in the mutant that could not grow on sucrose or trehalose in minimal medium, we have shown that* treB* and* treC* are involved in trehalose transport and metabolism. The reason for their transcription downregulation in the mutant is not known.

In addition to downregulation of the transcription of* treB* and* treC*, the qRT-PCR results confirmed microarray data indicating that the expression of the transcriptional antiterminator CPF_0069 was substantially downregulated in the mutant 13124^GR^ in comparison with the wild type. CPF_0069 codes for an RNA-binding protein similar to the BglG-like transcriptional antiterminators found in other bacteria [22, 37, 38]. CPF_0069 is located upstream of CPF_0070 and CPF_0071, which, respectively, encode the IIA and IICB components of a putative* N*-acetylglucosamine PTS. Microarray data showed that CPF_0070 and CPF_071 were also downregulated in the mutant and most likely are regulated by the associated antiterminator. The mutant therefore shows several defects in expression of phosphotransferases involved in uptake and phosphorylation of sugar substrates. However, the role that mutation in CPF_0069, or its downregulation, may have played in altering the expression of other genes in the resistant mutant is not known and merits further investigation.

Another PTS-associated mechanism of controlling the transcription of genes involves the catabolite control protein A (CcpA), a master transcription factor that regulates global gene transcription by binding to a catabolite response element (*cre*) when a readily-metabolizable carbohydrate is present [39]. This form of regulation is mediated through phosphorylation of the PTS phosphocarrier protein HPr at amino acid Ser46 by the metabolite-activated protein kinase, HPrK. HPr(ser)P then forms a complex with CcpA, resulting in stimulation of DNA binding. The coding regions of these proteins were identical in the wild type ATCC 13124 and mutant 13124^GR^, and there was less than a twofold difference between the levels of transcription of HPrK (CPF_1261) and CcpA (CPF_2683) in the wild type and mutant (data not shown). The role of these proteins, if any, in trehalose transport in the mutant is not known at this time.

In addition to the regulatory genes associated with the PTS, there are other regulatory genes in* C. perfringens*, notably the two-component transduction (sensor/regulator) system* VirR/virS* that regulates a variety of genes, including the genes for regulatory RNA (*vrr*) and several toxins [40, 41].* vrr* is a global response regulatory RNA (VR-RNA), transcription of which is regulated by VirR/VirS through a complex regulatory network via a VirR/VirS-VR-RNA cascade, and its transcript VR-RNA, rather than a protein, has regulatory activity [40, 41].* vrr* was substantially downregulated in the mutant. Using next-generation sequencing, a mutation was found in* virS *of* C. perfringens* strain 13124^GR^ that resulted in introduction of a stop codon terminating protein synthesis after 18 amino acids. This change, previously undetected [28], was confirmed by PCR and sequencing and may have resulted in changes in transcription of various genes, including the downregulation of* vrr*. Ohtani et al. [41] found downregulation of genes, including PTS genes (CPE1463–1466), the toxin gene* plc* (CPE0036) and one antiterminator gene (CPE2553), in a* vrr *deletion mutant of* C. perfringens *strain 13. Although the downregulated PTS genes and antiterminator gene in their study were different from those we found in the fluoroquinolone-resistant mutant 13124^GR^, it indicates that downregulation of* vrr* may affect the PTS in* C. perfringens. *The effect of downregulation of* vrr *observed in this study on disaccharide transport needs further investigation.

Addition of trehalose to cultures of* C. perfringens* ATCC 13124 improved the tolerance of the cultures to increased salt concentration. The growth rate of the wild type was similar in minimal medium supplemented with different sugars, with or without 0.5% NaCl. When the salt concentration was increased to 1%, the growth rate of cells in cultures containing glucose was 0.076 h^−1^, much lower than the growth rate in cultures containing either sucrose (0.1235 h^−1^) or trehalose (0.1335 h^−1^). In cultures containing 2% NaCl, growth was observed only in cultures containing trehalose or sucrose. This indicates that sucrose and trehalose protected the cells from NaCl and trehalose was more protective. Sucrose is known to protect* C. perfringens* during L-form formation [17], but this is the first time that the effect of trehalose on the protection of* C. perfringens* from NaCl has been shown.

In conclusion, the study shows that trehalose can protect* C. perfringens* from environmental stress. Furthermore, CPF_0541, annotated as* treB*, is implicated in trehalose transport, and its expression is altered in the gatifloxacin-resistant mutant 13124^GR^.

## Figures and Tables

**Table 1:** Primers designed for the amplification of trehalose operon genes in *C. perfringens *along with the upstream regions.

Name	Gene	Sequence	Location	Size
CPF_0539F	Sigma-70 factor	TGAGTTCCAAAATAGACAAGAT	640794–640815	22-mer
CPF_0539R	Sigma-70 factor	CCCCATAAGCCAAGGTG	642208–642224	17-mer
CPF_0540F	Hypothetical protein	AATGGGGTAGGAATAACTTTGTCA	642326–642349	24-mer
CPF_0540R	Hypothetical protein	CTTTTCCGCCTATGTACTCTGTTA	643493–643516	24-mer
1985F	CPF_0540 upstream	ACAAGCTTTAGGTGAGGTTT	641985–642004	20-mer
tre373F	*treB*	TGATATGAATAAGGAGGGTGTTGG	642793–642816	24-mer
tre1027F	*treB*	TATGGAGGGAAAAAGATGAGTAAG	643447–643470	24-mer
tre2172R	*treB*	TGCTGGTATTGATATTTGTTTTTC	644569–644592	24-mer
tre1920F	*treB*	TATAGTGGCAGGCTTAACATCATC	644340–644363	24-mer
tre3128R	*treC*	CTCTCCTAGCTCCTCAACATACTC	645525–645548	24-mer
tre3090F	*treC*	GGAGGGAATGCTTGGGAGTA	645510–645529	20-mer
tre3777R	*treC*	AACCTGGATTTGTCATTC	646180–646197	18-mer
tre3688F	*treC*	CTTCAAAAATGCTAGCGACTTCAA	646108–646131	24-mer
tre4667R	*treC-treR*	TGCTTCAAAAATTCCACTATCTA	647065–647087	23-mer
tre4406F	*treR*	AAATTAAGAAGGGGTTGTAT	646826–646845	20-mer
tre5283R	*treR*	ACTTAAATTTATCTGGTCTATGTC	647680–647703	24-mer
treRR	*treR*	CTTTAATAGCTGGTGATAGTT	648367–648387	21-mer

**Table 2:** Comparison of the gene transcription in wild type *C. perfringens *ATCC 13124 and gatifloxacin resistant mutant 13124^GR^ by qRT-PCR^a^.

Locus	Gene	Primer	Fold (mutant/wild)		
CPF_0541 upstream	*treB* upstream	ACAGAGTACATAGGCGGAAAAGA	−333.89	−828.5	−330
		TTTCAACATCTGCTTTGCTTG			
CPF_0541	*treB*	TTGGGACTTTGGATTTGCTC	−93.81	−227.5	−22.28
		GCCTGCCACTATAGCTCCTG			
CPF_0542	*treC*	GAGCTTTAAAGGGCGAAGAAA	−45.1	−44.50	−16.80
		CCCAGTTTAAATCAGCCTGTG			
CPF_0543	*treR*	GCCTTCAGAATCACAACTTATGG	−1.69	−1.79	−1.31
		TTTCGCACTCTTTTCCTAAGC			
CPF_0539	RNA polymerase sigma-70 factor	TGTTGGATGAAAGTACACCGA	2.29	3.63	2.99
		AGCCCTCCTTTAAAACCTCA			
CPF_0069	Transcription antiterminator	ATTCGGCAAGAACAACAGGA	−90.74	−711.9	−116.6
		GCAACCTTAAAGGATTCTGGA			
CPF_0042	*plc*	TGACACAGGGGAATCACAAA	−9.58	−14.20	
		CGCTATCAACGGCAGTAACA			
CPF_0623	*revR*	AGTCCTAATAGTAGATGATGAGGA	−1.92	−1.73	−1.51
		CTTATAAGCTTTAGAGCTTCAGT			
CPF_0007	*gyrA*	TGCCAGAATAGTTGGGGAAG	1.08	1.27	
		TACCATGTCCGTCAACAAGC			
CPF_1204	*vrr*	CAAAAAGGATTTTAACAAGTGCAA	−596.80	−133.1	
		TTGATATTAAAGCAAGTATGGGACT			
CPF_1956	*topA*	ACCTTGCAACTGACCCTGAT	−2.38	−2.42	−2.07
		CAGCACTCAATCCCCATTTT			
CPF_1760	*topB*	AACTTGGGCTTTAGGCCATT	−1.61		
		GCTACGAGCTCCCCTTCTCT			
CPF_1785	PTS sucrose-specific IIBC component	GCGGCCATATTTGGAGTAAA	−92.15		
		CCAGTTCCAGCCAATTTCAT			

^a^ *treB* and *treC* are trehalose-specific genes. The transcription of several genes was considerably decreased in the mutant 13124^GR^ in comparison with the wild type ATCC 13124; the data presented represent those obtained in different experiments. The expression of topoisomerase genes (*gyr *and *top*) and response regulator gene (*revR*) was not markedly changed. *Pfo,* which was also downregulated, was reported previously [28].

## References

[1] Cheung J. K., Wisniewski J. A., Adams V. M., Quinsey N. S., Rood J. I. (2016). Analysis of the virulence-associated RevSR two-component signal transduction system of *Clostridium perfringens*. *International Journal of Medical Microbiology*.

[2] Myers G. S. A., Rasko D. A., Cheung J. K. (2006). Skewed genomic variability in strains of the toxigenic bacterial pathogen, *Clostridium perfringens*. *Genome Research*.

[3] Ohtani K., Shimizu T. (2015). Regulation of toxin gene expression in *Clostridium perfringens*. *Research in Microbiology*.

[4] Strom A. R., Kaasen I. (1993). Trehalose metabolism in *Escherichia coli*: stress protection and stress regulation of gene expression. *Molecular Microbiology*.

[5] Leslie S. B., Israeli E., Lighthart B., Crowe J. H., Crowe L. M. (1995). Trehalose and sucrose protect both membranes and proteins in intact bacteria during drying. *Applied and Environmental Microbiology*.

[6] Kempf B., Bremer E. (1998). Uptake and synthesis of compatible solutes as microbial stress responses to high-osmolality environments. *Archives of Microbiology*.

[7] Sleator R. D., Hill C. (2002). Bacterial osmoadaptation: the role of osmolytes in bacterial stress and virulence. *FEMS Microbiology Reviews*.

[8] Elbein A. D., Pan Y. T., Pastuszak I., Carroll D. (2003). New insights on trehalose: a multifunctional molecule. *Glycobiology*.

[9] Cardoso F. S., Castro R. F., Borges N., Santos H. (2007). Biochemical and genetic characterization of the pathways for trehalose metabolism in *Propionibacterium freudenreichii*, and their role in stress response. *Microbiology*.

[10] Giaever H. M., Styrvold O. B., Kaasen I., Strøm A. R. (1988). Biochemical and genetic characterization of osmoregulatory trehalose synthesis in *Escherichia coli*. *Journal of Bacteriology*.

[11] Mikkat S., Effmert U., Hagemann M. (1997). Uptake and use of the osmoprotective compounds trehalose, glucosylglycerol, and sucrose by the *Cyanobacterium synechocystis* sp. PCC6803. *Archives of Microbiology*.

[12] Makihara F., Tsuzuki M., Sato K. (2005). Role of trehalose synthesis pathways in salt tolerance mechanism of *Rhodobacter sphaeroides* f. sp. *denitrificans* IL106. *Archives of Microbiology*.

[13] Purvis J. E., Yomano L. P., Ingram L. O. (2005). Enhanced trehalose production improves growth of *Escherichia coli* under osmotic stress. *Applied and Environmental Microbiology*.

[14] Barra L., Pica N., Gouffi K., Walker G. C., Blanco C., Trautwetter A. (2003). Glucose 6-phosphate dehydrogenase is required for sucrose and trehalose to be efficient osmoprotectants in *Sinorhizobium meliloti*. *FEMS Microbiology Letters*.

[15] Jain N. K., Roy I. (2009). Effect of trehalose on protein structure. *Protein Science*.

[16] Zhao P., Zhou Z., Zhang W., Lin M., Chen M., Wei G. (2015). Global transcriptional analysis of *Escherichia coli* expressing IrrE, a regulator from *Deinococcus radiodurans*, in response to NaCl shock. *Molecular BioSystems*.

[17] Mahony D. E., Mader J. A., Dubel J. R. (1988). Transformation of *Clostridium perfringens* L forms with shuttle plasmid DNA. *Applied and Environmental Microbiology*.

[18] Rimmele M., Boos W. (1994). Trehalose-6-phosphate hydrolase of *Escherichia coli*. *Journal of Bacteriology*.

[19] Carvalho A. L., Cardoso F. S., Bohn A., Neves A. R., Santos H. (2011). Engineering trehalose synthesis in *Lactococcus lactis* for improved stress tolerance. *Applied and Environmental Microbiology*.

[20] Gao Y., Xi Y., Lu X.-L. (2013). Cloning, expression and functional characterization of a novel trehalose synthase from marine *Pseudomonas* sp. P8005. *World Journal of Microbiology & Biotechnology*.

[21] Maréchal L. R. (1984). Transport and metabolism of trehalose in *Escherichia coli* and *Salmonella typhimurium*. *Archives of Microbiology*.

[22] Mitchell W. J. (2015). The phosphotransferase system in solventogenic clostridia. *Journal of Molecular Microbiology and Biotechnology*.

[23] Mitchell W. J. (2016). Sugar uptake by the solventogenic clostridia. *World Journal of Microbiology & Biotechnology*.

[24] Shimizu T., Ohtani K., Hirakawa H. (2002). Complete genome sequence of *Clostridium perfringens*, an anaerobic flesh-eater. *Proceedings of the National Academy of Sciences of the United States of America*.

[25] Bhumiratana A., Anderson R. L., Costilow R. N. (1974). Trehalose metabolism by *Bacillus popilliae*. *Journal of Bacteriology*.

[26] Bürklen L., Schöck F., Dahl M. K. (1998). Molecular analysis of the interaction between the *Bacillus subtilis* trehalose repressor TreR and the tre operator. *Molecular & General Genetics*.

[27] Dahl M. K. (1997). Enzyme II^Glc^ contributes to trehalose metabolism in *Bacillus subtilis*. *FEMS Microbiology Letters*.

[28] Park S., Park M., Rafii F. (2013). Comparative transcription analysis and toxin production of two fluoroquinolone-resistant mutants of *Clostridium perfringens*. *BMC Microbiology*.

[29] Rashid M., Weintraub A., Nord C. E. (2011). Comparative effects of the immediate and the extended release formulations of ciprofloxacin on normal human intestinal microflora. *Journal of Chemotherapy*.

[30] Komp Lindgren P., Marcusson L. L., Sandvang D., Frimodt-Møller N., Hughes D. (2005). Biological cost of single and multiple norfloxacin resistance mutations in *Escherichia coli* implicated in urinary tract infections. *Antimicrobial Agents and Chemotherapy*.

[31] Kugelberg E., Löfmark S., Wretlind B., Andersson D. I. (2005). Reduction of the fitness burden of quinolone resistance in *Pseudomonas aeruginosa*. *Journal of Antimicrobial Chemotherapy*.

[32] Park M., Sutherland J. B., Kim J. N., Rafii F. (2013). Effect of fluoroquinolone resistance selection on the fitness of three strains of *Clostridium perfringens*. *Microbial Drug Resistance*.

[33] Rafii F., Park M., Gamboa da Costa G., Camacho L. (2009). Comparison of the metabolic activities of four wild-type *Clostridium perfringens* strains with their gatifloxacin-selected resistant mutants. *Archives of Microbiology*.

[34] Park M., Rafii F. (2014). Global phenotypic characterization of effects of fluoroquinolone resistance selection on the metabolic activities and drug susceptibilities of *Clostridium perfringens* strains. *International Journal of Microbiology*.

[35] Rafii F., Park M., Novak J. S. (2005). Alterations in DNA gyrase and topoisomerase IV in resistant mutants of *Clostridium perfringens* found after in vitro treatment with fluoroquinolones. *Antimicrobial Agents and Chemotherapy*.

[36] Sebald M., Costilow R. N. (1975). Minimal growth requirements for *Clostridium perfringens* and isolation of auxotrophic mutants. *Applied Microbiology*.

[37] Tangney M., Mitchell W. J. (2007). Characterisation of a glucose phosphotransferase system in *Clostridium acetobutylicum* ATCC 824. *Applied Microbiology and Biotechnology*.

[38] Al Makishah N. H., Mitchell W. J. (2013). Dual substrate specificity of an N-acetylglucosamine phosphotransferase system in *Clostridium beijerinckii*. *Applied and Environmental Microbiology*.

[39] Deutscher J., Francke C., Postma P. W. (2006). How phosphotransferase system-related protein phosphorylation regulates carbohydrate metabolism in bacteria. *Microbiology and Molecular Biology Reviews*.

[40] Okumura K., Ohtani K., Hayashi H., Shimizu T. (2008). Characterization of genes regulated directly by the VirR/VirS system in *Clostridium perfringens*. *Journal of Bacteriology*.

[41] Ohtani K., Hirakawa H., Tashiro K., Yoshizawa S., Kuhara S., Shimizu T. (2010). Identification of a two-component VirR/VirS regulon in *Clostridium perfringens*. *Anaerobe*.

